# Supplementary material for: Unveiling the Bioactive Potential of the Invasive Jellyfish Phyllorhiza punctata Through Integrative Transcriptomic and Proteomic Analyses
Source: Biomolecules. 2025 Aug 4;15(8):1121. doi: 10.3390/biom15081121 (PMC12383608; doi:10.3390/biom15081121)
Supplement: Supplementary file 1 [file biomolecules-15-01121-s001.zip › Suplementary Material/FileS3/PEPFOLD4/XP_065057987_8463/clusters.html]

 PEP-FOLD clusters


Download archive of all models  here   

|  |  |  |
| --- | --- | --- |
| # | file | sOPEP |
| # cluster 1 - Size: 8 |  |  |
|  | XP\_065057987\_8463-model1 | -8.68442 |
|  | XP\_065057987\_8463-model1.1 | -8.68442 |
|  | XP\_065057987\_8463-model1.2 | -8.61757 |
|  | XP\_065057987\_8463-model1.3 | -8.61757 |
|  | XP\_065057987\_8463-model1.4 | -8.61757 |
|  | XP\_065057987\_8463-model1.5 | -8.59583 |
|  | XP\_065057987\_8463-model1.6 | -8.54671 |
|  | XP\_065057987\_8463-model1.7 | -8.54671 |
| # cluster 2 - Size: 45 |  |  |
|  | XP\_065057987\_8463-model2 | -5.51719 |
|  | XP\_065057987\_8463-model2.1 | -5.51719 |
|  | XP\_065057987\_8463-model2.2 | -5.51719 |
|  | XP\_065057987\_8463-model2.3 | -5.51719 |
|  | XP\_065057987\_8463-model2.4 | -5.51719 |
|  | XP\_065057987\_8463-model2.5 | -5.51719 |
|  | XP\_065057987\_8463-model2.6 | -5.51719 |
|  | XP\_065057987\_8463-model2.7 | -5.44142 |
|  | XP\_065057987\_8463-model2.8 | -5.44142 |
|  | XP\_065057987\_8463-model2.9 | -5.44142 |
|  | XP\_065057987\_8463-model2.10 | -5.44142 |
|  | XP\_065057987\_8463-model2.11 | -5.44142 |
|  | XP\_065057987\_8463-model2.12 | -5.44142 |
|  | XP\_065057987\_8463-model2.13 | -5.32514 |
|  | XP\_065057987\_8463-model2.14 | -5.32514 |
|  | XP\_065057987\_8463-model2.15 | -5.32514 |
|  | XP\_065057987\_8463-model2.16 | -5.32452 |
|  | XP\_065057987\_8463-model2.17 | -5.32452 |
|  | XP\_065057987\_8463-model2.18 | -5.32452 |
|  | XP\_065057987\_8463-model2.19 | -5.32452 |
|  | XP\_065057987\_8463-model2.20 | -5.32452 |
|  | XP\_065057987\_8463-model2.21 | -5.28801 |
|  | XP\_065057987\_8463-model2.22 | -5.28177 |
|  | XP\_065057987\_8463-model2.23 | -5.28177 |
|  | XP\_065057987\_8463-model2.24 | -5.21248 |
|  | XP\_065057987\_8463-model2.25 | -5.21248 |
|  | XP\_065057987\_8463-model2.26 | -5.21248 |
|  | XP\_065057987\_8463-model2.27 | -5.21248 |
|  | XP\_065057987\_8463-model2.28 | -5.21248 |
|  | XP\_065057987\_8463-model2.29 | -5.21248 |
|  | XP\_065057987\_8463-model2.30 | -5.21248 |
|  | XP\_065057987\_8463-model2.31 | -5.15774 |
|  | XP\_065057987\_8463-model2.32 | -5.11601 |
|  | XP\_065057987\_8463-model2.33 | -5.09906 |
|  | XP\_065057987\_8463-model2.34 | -5.09906 |
|  | XP\_065057987\_8463-model2.35 | -5.09906 |
|  | XP\_065057987\_8463-model2.36 | -5.09906 |
|  | XP\_065057987\_8463-model2.37 | -5.09906 |
|  | XP\_065057987\_8463-model2.38 | -5.09906 |
|  | XP\_065057987\_8463-model2.39 | -5.09906 |
|  | XP\_065057987\_8463-model2.40 | -5.09906 |
|  | XP\_065057987\_8463-model2.41 | -5.09307 |
|  | XP\_065057987\_8463-model2.42 | -5.07256 |
|  | XP\_065057987\_8463-model2.43 | -4.98236 |
|  | XP\_065057987\_8463-model2.44 | -4.81248 |
| # cluster 3 - Size: 12 |  |  |
|  | XP\_065057987\_8463-model3 | -5.4409 |
|  | XP\_065057987\_8463-model3.1 | -5.35363 |
|  | XP\_065057987\_8463-model3.2 | -5.28549 |
|  | XP\_065057987\_8463-model3.3 | -5.28549 |
|  | XP\_065057987\_8463-model3.4 | -5.28549 |
|  | XP\_065057987\_8463-model3.5 | -5.28549 |
|  | XP\_065057987\_8463-model3.6 | -5.0959 |
|  | XP\_065057987\_8463-model3.7 | -5.0959 |
|  | XP\_065057987\_8463-model3.8 | -5.08939 |
|  | XP\_065057987\_8463-model3.9 | -5.05224 |
|  | XP\_065057987\_8463-model3.10 | -4.97339 |
|  | XP\_065057987\_8463-model3.11 | -4.90631 |
| # cluster 4 - Size: 35 |  |  |
|  | XP\_065057987\_8463-model4 | -5.38055 |
|  | XP\_065057987\_8463-model4.1 | -5.38055 |
|  | XP\_065057987\_8463-model4.2 | -5.38055 |
|  | XP\_065057987\_8463-model4.3 | -5.31513 |
|  | XP\_065057987\_8463-model4.4 | -5.31513 |
|  | XP\_065057987\_8463-model4.5 | -5.31513 |
|  | XP\_065057987\_8463-model4.6 | -5.31513 |
|  | XP\_065057987\_8463-model4.7 | -5.31513 |
|  | XP\_065057987\_8463-model4.8 | -5.29244 |
|  | XP\_065057987\_8463-model4.9 | -5.29244 |
|  | XP\_065057987\_8463-model4.10 | -5.29244 |
|  | XP\_065057987\_8463-model4.11 | -5.29244 |
|  | XP\_065057987\_8463-model4.12 | -5.29244 |
|  | XP\_065057987\_8463-model4.13 | -5.27361 |
|  | XP\_065057987\_8463-model4.14 | -5.27361 |
|  | XP\_065057987\_8463-model4.15 | -5.27361 |
|  | XP\_065057987\_8463-model4.16 | -5.27361 |
|  | XP\_065057987\_8463-model4.17 | -5.27361 |
|  | XP\_065057987\_8463-model4.18 | -5.27361 |
|  | XP\_065057987\_8463-model4.19 | -5.27361 |
|  | XP\_065057987\_8463-model4.20 | -5.2261 |
|  | XP\_065057987\_8463-model4.21 | -5.2261 |
|  | XP\_065057987\_8463-model4.22 | -5.2261 |
|  | XP\_065057987\_8463-model4.23 | -5.2122 |
|  | XP\_065057987\_8463-model4.24 | -5.2122 |
|  | XP\_065057987\_8463-model4.25 | -5.2122 |
|  | XP\_065057987\_8463-model4.26 | -5.12772 |
|  | XP\_065057987\_8463-model4.27 | -5.09638 |
|  | XP\_065057987\_8463-model4.28 | -5.09638 |
|  | XP\_065057987\_8463-model4.29 | -4.99528 |
|  | XP\_065057987\_8463-model4.30 | -4.98912 |
|  | XP\_065057987\_8463-model4.31 | -4.92132 |
|  | XP\_065057987\_8463-model4.32 | -4.88185 |
|  | XP\_065057987\_8463-model4.33 | -4.84216 |
|  | XP\_065057987\_8463-model4.34 | -4.21245 |
|  |  |  |
